# What Can Eye Movements Tell Us about Higher Level Comprehension?

**DOI:** 10.3390/vision3030045

**Published:** 2019-09-06

**Authors:** Anne E. Cook, Wei Wei

**Affiliations:** Department of Educational Psychology, University of Utah, Salt Lake City, UT 84112, USA

**Keywords:** eye tracking, reading, anaphor, discourse comprehension

## Abstract

The majority of eye tracking studies in reading are on issues dealing with word level or sentence level comprehension. By comparison, relatively few eye tracking studies of reading examine questions related to higher level comprehension in processing of longer texts. We present data from an eye tracking study of anaphor resolution in order to examine specific issues related to this discourse phenomenon and to raise more general methodological and theoretical issues in eye tracking studies of discourse processing. This includes matters related to the design of materials as well as the interpretation of measures with regard to underlying comprehension processes. In addition, we provide several examples from eye tracking studies of discourse to demonstrate the kinds of questions that may be addressed with this methodology, particularly with respect to the temporality of processing in higher level comprehension and how such questions correspond to recent theoretical arguments in the field.

## 1. Introduction

The use of eye tracking technology to study reading has roots in early work on physical movements of the eyes during reading, e.g., [1,2,3,4,5], as well as on the properties of the perceptual span, which refers to the area of visual acuity within a single fixation, e.g., [6,7]. Subsequent work focused on how studying individuals’ eye movements during reading can provide information about underlying processes, such as those involved in word recognition, semantic access, syntactic parsing, and higher level comprehension. Although there have been a large number of studies that have used eye movements to examine word level and sentence level processing during reading (for reviews of that work, see, e.g., [8,9,10]), there have been far fewer studies that have applied eye tracking technology to the study of higher level comprehension. This latter issue is the topic of this article.

One primary reason that higher level comprehension has received less attention in eye tracking research on reading is that the texts necessary to study processing at this level are longer and, thus, more complex to construct than the sentence-level texts typically used for studies on word- and sentence-level processing. Most eye tracking studies of reading examine eye movement measures for a targeted word or short phrase that is embedded within a sentence; processing time on these regions is dependent upon properties of the word/phrase itself and/or properties of the immediately preceding information; see [11]. This is not meant to imply that the word and sentence level studies are limited to single-sentence stimuli; several studies have employed paragraph-level texts to study these processes, e.g., [12,13,14]. However, the great majority of stimuli used to study word- and sentence-level processes are composed of one to a few sentences.

Since higher level comprehension entails integrating and validating information across multiple sentences, paragraphs, or even texts, the materials that are used to study processes that contribute to higher level comprehension are necessarily different in nature. In addition to being longer, the nature of what is being studied is different; researchers may be interested in issues such as whether paragraph contexts support activation of particular inferences, or how and when readers process inconsistencies in information from various points of a text. Thus, it is a more complex task to design paragraph-level stimuli such that the evidence for the type of higher level processing under investigation can be isolated to a particular word or phrase. For example, in self-paced reading studies that employ the inconsistency paradigm to investigate reactivation of information from memory during reading, materials are constructed so that a target sentence is either consistent or inconsistent with respect to previous information presented in the passage [15]. For example, in [15], the target sentence “Mary ordered a cheeseburger and fries” can be consistent with the prior context about her being a junk food lover, but inconsistent with the context about her being a strict vegetarian. Participants in these studies advance through each passage line by line, and reading time for the entire target sentence is the dependent measure, which is predicted to vary as a function of the preceding context. In eye tracking studies, however, it is important to be able to isolate the critical region of text to a single word or short phrase. With longer target regions of text, the researcher runs the risk that processing difficulty will be distributed across multiple words, thus diluting the ability to determine where processing difficulty begins and ends and how those effects play out over time. Eye tracking researchers interested in discourse processing must be careful to select and design their materials carefully; it is not advisable to just assume that materials created for line-by-line studies can be readily used in eye tracking studies. We will argue that careful construction of stimulus materials is essential to the investigation of critical issues that are at the crux of theoretical debates in discourse processing. 

Another pragmatic issue in using eye tracking to study higher level comprehension concerns the use and interpretation of the specific measures that are derived from the eye movement record. For example, studies of word-level processing are often focused on issues that impact processing very early on during reading, thus the measures that provide the most information are often those that reflect the earliest stages of processing a word (e.g., probability of skipping, single fixation duration, first fixation duration). The probability of skipping a word refers to the probability that the reader does not fixate on a word/region when moving from left to right across a text. Single fixation duration is the duration of a fixation if only one fixation is made on a word before the reader moves past it, and first fixation duration is the duration of the first fixation made on a word. Single fixation duration and first fixation duration are highly correlated; they only differ when a reader makes multiple fixations on a word before moving past it in the text. Researchers may also report other measures that reflect ongoing processing of the word and subsequent integration of it with the surrounding sentence/passage context (i.e., first-pass duration, go-past duration). First-pass duration is defined as the amount of time from when a reader first fixates on a region to when they first leave that region, whereas go-past duration (also sometimes referred to as regression path duration) is the amount of time from when a reader first fixates on a region to when they first leave that region to the right. First pass and go-past duration differ when readers leave a region to regress back to and reread previous content; go-past includes the time spent rereading whereas first-pass duration does not. For a more detailed discussion of these measures, see the overview provided by Cook and Wei [11]. Higher level comprehension reflects processes that occur somewhat downstream in the time course from word recognition and lexical access, so the most useful measures for studies in this area also tend to reflect processing that occurs later in the time course of reading (i.e., first-pass duration, go-past duration) as well as attempts to review/reread previous material in an attempt to resolve processing difficulty. 

Across all levels of processing, it is also important to examine the extent to which readers reread previously presented portions of text in attempts to resolve comprehension difficulty. Researchers often report information about regressions, or eye movements made to previously encountered material; regressions are typically reported in terms of the probability of regressing into a region of text or the probability of regressing out of a region of text. Another commonly reported measure is rereading, or second-pass duration, which includes all refixations on a region of text after the eye has already moved past that region in the text. Many researchers hesitate to report second-pass duration, though, because not every reader rereads every item in every experimental condition, so there are many empty cells in the resulting data matrix. Instead, there is a recent trend in the literature to report total duration, which is the sum of initial processing of a target region (i.e., first-pass duration) and any subsequent rereading of that region (i.e., second-pass duration). The problem with reporting and interpreting total duration as a measure of delayed processing is that it includes initial processing time as well. We feel a better alternative is to report second pass reading time, but then include convergent measures of rereading behavior, such as probability of regressions into and/or out of the target region [8]. 

How individual eye tracking measures are interpreted is also influenced by the type of texts that are used. In single sentence stimuli, the entirety of the text is available on a single line on the screen, and likely, in the reader’s working memory when the target region is encountered. In these cases, readers often regress out of problematic content to reread preceding information. Thus, measures of rereading reflect not only probability of regressing out of the target region, but also regressions to earlier content, and rereading of both earlier content and the target region. In contrast, in studies of discourse comprehension where stimuli consist of multiple lines/sentences of text, the information needed to resolve processing difficulty may be several sentences back and, thus, no longer available in working memory and well outside the range of the reader’s perceptual span. This may make planning regressions several lines back to specific content presented earlier in the text much more difficult (and much less likely) than if the entire text were available on a single line. However, just how far back in a text readers will regress to reread content in attempts to resolve difficulty when processing extended paragraph level texts has not been studied extensively in the discourse processing literature. The distance that readers will regress, and the amount of previous content that they will reread, is likely to differ from sentence-level to paragraph-level studies, meaning that the interpretation of measures that involve rereading should also differ between these types of studies. For example, eye tracking researchers focused on discourse-level phenomena tend to focus their analyses on probability of regressions out of a target region and back to the target region, as well as rereading of the target region, rather than on regressions to or rereading of specific information presented earlier in a text. 

We have just established that the use of longer passages of text in research on reading is important for the understanding of eye movements during higher level comprehension, especially when texts require readers to establish connections between incoming content and previously encountered information that may no longer be available in the reader’s working memory. One particular phenomenon in which such connections are essential to comprehension is the case of anaphoric references. An anaphor is a word or phrase that refers to previously encountered content (i.e., an antecedent). Much of the research on anaphoric references has focused on the processes through which the antecedent is reactivated after the reader encodes the anaphoric reference. Studies on this topic have primarily utilized self-paced line-by-line reading paradigms, paired with probe response methodologies (e.g., [16,17,18]). Although early work in this area focused on questions concerning the processes governing reactivation of the antecedent, more recent work has focused on questions about what happens after the reactivation process. That is, what happens in anaphor processing after an antecedent has been reactivated? How are the two concepts integrated with and validated against one another, and how does this play out over time? 

Since anaphoric references are typically single words or short phrases, the study of anaphoric processing is ideally suited to eye tracking. However, relatively few eye tracking studies in this area have been conducted. O’Brien and colleagues [19,20] used eye tracking to demonstrate that ease of processing anaphoric references depends on the strength of the connection between the anaphor and its referent, as well as on the nature of the anaphoric phrase itself. However, the goal of those studies was to explore the antecedent reactivation process, not necessarily the time course of processing the anaphor. However, Duffy and Rayner [21] and Ehrlich and Rayner [22] found that processing difficulty on the text immediately following the anaphor was a function of the relation between the anaphor and its antecedent. This means that processing of the anaphor was not complete even when readers’ eyes had moved past it in the text. Thus, although researchers have long assumed that establishing antecedents for anaphors is “necessary” for comprehension [23], it may be that anaphoric processing is not as straightforward as originally assumed. This raises a critical question for anaphoric processing: is full reactivation of an antecedent required for successful comprehension of an anaphor, or is initial processing of the anaphor based on the goodness of fit of reactivated content with the anaphor? 

Although there has been research on incomplete processing, or shallow, or “good enough” processing in other domains [24,25], there has been little done in the realm of anaphoric processing, and to our knowledge, none with eye tracking. Most work in the area of shallow processing has been conducted with materials in which anomalous information replaced correct content in sentences or short paragraphs, and participants were explicitly asked to detect the anomalies. Researchers have consistently found that participants are less likely to detect anomalies when they are highly-related to the correct content than when they are low-related [24,25]. Putting this in the context of anaphor processing, Cook [26] argued that if processing of anaphors is not complete before readers move on in the text [21,22], then highly-related, but incorrect anaphors may be less likely to cause processing difficulty than low-related incorrect anaphors. If, on the other hand, full activation and resolution of anaphors is required for comprehension, the semantic relation between the anaphor and the antecedent should not matter if an anaphor is an incorrect referent for the antecedent. Cook [26] tested these arguments with a self-paced line-by-line reading paradigm in which she asked participants to read passages in which an anaphor (e.g., cello) was either correct with respect to an antecedent presented several sentences earlier in the passage (e.g., cello), incorrect but highly-related to the antecedent (e.g., violin), or incorrect and lowly-related to the antecedent (e.g., oboe). Across multiple experiments, Cook found that reading times on the target line containing the anaphor were a function of the semantic overlap between the anaphor and the antecedent, and that this processing difficulty played out across multiple sentences. Participants’ reading times on the target sentence were faster in the correct condition than in the incorrect conditions, and they were faster in the incorrect-high overlap condition than in the incorrect-low overlap condition. By the time participants reached the next sentence, the difference between the two incorrect conditions was no longer significant, although reading times in both conditions were still slower than in the correct condition. Cook suggested that initial reading times on the anaphor may have been based on their goodness of fit with reactivated information about the antecedent, thereby supporting an incomplete processing account of anaphor resolution. Additionally, consistent with the argument that processing of anaphors continues even after the eyes move past it in the text [21,22], incorrect anaphors influenced processing of information in the text after readers had moved past the line containing the anaphor. However, as discussed earlier, in the line-by-line reading paradigm, the unit of analysis is time to read an entire line; thus, it is not clear whether the processing difficulty on the target line in the incorrect conditions occurred on the anaphor itself, or after a delay. In addition, when reading line-by-line, readers are not able to regress back to previously encountered content to resolve comprehension difficulties. 

In a follow-up to Cook [26], Rayner and colleagues [27] varied whether anaphors were consistent or inconsistent with respect to their antecedents, as well as the distance between the anaphor and the antecedent. They used eye tracking to measure processing on the anaphor and found that when the anaphor was near the antecedent in the text (i.e., in adjacent sentences), readers spent more time processing incorrect anaphors and were more likely to regress back to the antecedent. When the anaphor was more distant (i.e., several sentences after the antecedent), however, there was no reliable effect of inconsistency on either time spent processing the anaphor or probability of regressing back to the antecedent. This suggests that comprehension of anaphors depends more on what information may be available in memory when the anaphor is encountered than on what the reader physically has access to in the text. The goal of the present study was to provide an additional test of the incomplete processing account in anaphor resolution by conducting an extension of Cook’s [26] and Rayner et al.’s [27] work. We used eye tracking to examine incomplete processing of anaphors, the timing of anaphoric processing (i.e., immediate or delayed), and the nature of information used to resolve difficulties in anaphoric processing. 

With respect to the first question about whether anaphoric processing is incomplete, fixation times should replicate the pattern of times observed by Cook [26] and Rayner et al. [27]; reading times should be faster in the correct condition than in the incorrect conditions, and they should be faster in the incorrect-high overlap condition than in the incorrect-low overlap condition. This would also mean that processing difficulty due to incorrect anaphors may be most likely to occur after the reader moved past the anaphor [21,22]; differences as a function of condition would be observed only in measures that reflect delayed processing of the anaphor, such as rereading (i.e., second pass) of the anaphor and probability of regressing into the anaphor. 

With respect to the question about what information readers utilize in resolving comprehension difficulty due to incorrect anaphors, we examined regressions back to previous content from the target line. Although Rayner and colleagues [27] did measure the probability of regressing out of the anaphor and back to the antecedent, they only found significant effects of anaphor inconsistency in the near condition. It may be that readers regressed out of the anaphor when it was more distant from the antecedent, but their regressions never reached the antecedent itself. This suggests that anaphor resolution depends on reactivated information about the antecedent in working memory. Providing a more detailed analysis of readers’ regression behaviors during comprehension of passage-level texts will provide information about whether readers actually revisit the explicit mention of the antecedent in order to resolve comprehension difficulty, or whether they mostly utilize content that has been reactivated in working memory in response to the anaphor. There is considerable evidence in the research literature that readers do consult previously read information when processing difficult text. However, much of this comes from work on expository texts, or in looking at individual differences and/or reader strategies [28,29,30]. The present study examines the extent to which readers reread previously encountered content under “normal” reading demands during narrative comprehension, when there is no specific task or strategy imposed on them other than reading for understanding. 

## 2. Materials and Methods 

Participants. Twenty-four members of a large University community in the Northeastern United States participated in exchange for either money or course credit. 

Apparatus. Eye movements were recorded by a Fourward Technologies Inc. (San Marcos, TX, USA) Dual Purkinje Eye tracker that has a resolution of 10 min of arc. The eye tracker was interfaced with a computer that ran the experiment. Viewing was binocular, with eye location recorded from the right eye. The position of the participant’s eye was sampled every millisecond by the computer and averaged over four consecutive samples. The averaged horizontal and vertical positions of the eye were compared with those of the previous sample to determine whether the eye was fixated or moving. 

Passages were presented in their entirety on an NEC (Minato, Tokyo, Japan) 4FG monitor with up to 60 character spaces per line. During the experiment, the participant was seated 62 cm from the monitor, where four characters of text equaled 1° of visual angle. Luminance on the monitor was adjusted to a comfortable brightness for the participant, then held constant. The room was dark except for an indirect light source that enabled the experimenter to keep notes. 

Materials. The materials used were modified versions of the 24 passages from Cook [26]. An example appears in Table 1. Passages consisted of a brief introductory section, a context section that described an antecedent with one explicit and two implicit mentions, a transition sentence, and then a target sentence that contained an anaphoric reference to the antecedent. This anaphor was either a correct referent for the antecedent, incorrect but had high semantic overlap with the antecedent, or incorrect and had low semantic overlap with the antecedent. Note that the target sentence was exactly the same across all three conditions; the information about the antecedent was the only content that varied across conditions. Target sentences were positioned within the text such that the anaphor appeared in the middle of the sentence and did not appear at the beginning of a line of text, and several words followed the anaphor prior to the end of the sentence/line. The target region consisted of the anaphor; target regions ranged from five to nine characters, and were, on average, 6.58 characters in length (SD = 1.18). Passages ended with a brief closing sentence. Mean lengths of the passages for the correct, incorrect-high, and incorrect-low antecedent conditions were 96.88, 97.5, and 97.54 words, respectively. 

Three materials sets were constructed, such that each set contained eight passages that appeared in each of the three conditions. Across the three materials sets, each passage appeared once in each of the three conditions. Each set of 24 experimental passages always appeared intermixed with a set of 48 additional filler passages that were designed to mask the purpose of the experiment; of the 48 filler passages, 12 contained incorrect information (although not in anaphoric references), while the remaining 36 did not contain any incorrect content. Thus, across all experimental and filler passages, 28 items contained incorrect information, and 54 items did not contain any incorrect content.

Procedure. All participants gave their informed consent for inclusion before they participated in the study. The study was conducted in accordance with the Declaration of Helsinki, and the protocol was approved by the University’s Internal Review Board Committee (Protocol 13440). Each individual participated in a session that lasted approximately 60 min. For each participant, a clay bite bar was prepared to eliminate head movements, and the eye tracker was calibrated. The initial calibration procedure took approximately five minutes. Prior to reading each passage, calibration of the eye tracking system was checked to ensure that accurate records were obtained. Each participant read three practice passages followed by the set of 24 experimental and 48 filler passages. Participants were told that they would be reading a series of paragraphs displayed on a computer monitor. They were told to read for comprehension so that they would be able to answer an occasional “yes/no” oral comprehension question; comprehension questions focused on content from the passage other than the anaphor and appeared after one fourth of the passages. The comprehension question for the sample passage in Table 1 was “Was Terry with her friend Jill?” At the beginning of each trial, five boxes appeared across the top of the screen, one box appeared in the middle, and five boxes appeared at the bottom of the screen. Each participant was instructed to look at the middle box until the experimenter said, “Ready,” and then to look at the left-most box. Once the experimenter had determined that the participant was fixating on the left-most box, the entire passage was presented on the screen. When the participant was finished reading the passage, he or she was instructed to press a button that would end the trial. Participants were given a brief break approximately halfway through the experiment. 

## 3. Results

Across all analyses, F_1_ and t_1_ indicate analyses based on participants variability and F_2_ and t_2_ indicate analyses based on items variability. All contrasts were significant at the *p* < 0.05 level, unless otherwise indicated.

Overall, comprehension question accuracy was high, with a mean of 85%; there was no difference in accuracy across the three experimental conditions, Fs < 1. In addition, there was no change in the size of the effect of antecedent condition from the beginning to the end of the experiment, Fs < 1. This was also true for all additional analyses reported below, Fs < 1; the size of the effect did not change over the course of the experiment, suggesting that readers’ reactions to or strategies for processing incorrect content did not change with multiple exposures. 

Mean first-pass reading time, go-past reading time, and second-pass reading times for the anaphor are reported in Table 2, as well as the mean probability of regressions into the anaphor from subsequent text. Consistent with the argument that individual fixations below 100 ms and above 1000 ms are uncommon and more likely to reflect measurement error [8], any fixations outside this range were excluded from the analysis. Any other outliers more than three standard deviations beyond the cell mean were excluded from analysis; this resulted in the elimination of less than 2% of the data. 

Antecedent condition had no impact on measures that reflect initial processing on the anaphor (i.e., first pass), or before the eyes moved past it (i.e., go-past), all Fs < 1. In addition, there was no effect of the antecedent condition on the probability of regression out of the antecedent, all Fs < 1. Thus, it must be the case that Cook’s [26] results were due to processing that took place after readers had moved past the anaphor in the target sentence. Consistent with this, the main effect of antecedent condition on second pass reading times was significant for the anaphor, F_1_(2, 46) = 5.43, MSe = 3193, *p* = 0.008, partial η^2^ = 0.19; F_2_(2, 46) = 6.67, MSe = 2325, *p* = 0.003, partial η^2^ = 0.23. Second-pass reading times on the anaphor were faster in the correct condition than in both the incorrect-high overlap condition, F_1_(1, 23) = 5.95, MSe = 3589, d = 0.68; F_2_(1, 23) = 7.71, MSe = 2381, d = 0.83, and the incorrect-low overlap condition, F_1_(1, 23) = 8.12, MSe = 8517, d = 0.88; F_2_(1, 23) = 16.3, MSe = 3799, d = 1.08, but the difference between the two incorrect-overlap conditions was not significant, F_1_(1, 23) = 1.93, *p* = 0.18, MSe = 7056; F_2_(1, 23)= 1.65, *p* = 0.21, MSe = 7772. 

In addition, the main effect of antecedent condition was significant for probability of regressions to the anaphor, F_1_(2, 46) = 4.82, MSe = 124, *p* = 0.01, partial η^2^ = 0.17; F_2_(2, 46) = 3.68, MSE = 145.98, *p* = 0.03, partial η^2^ = 0.14. Readers regressed to the anaphor less often in the correct antecedent condition than in either the incorrect-high overlap condition, F_1_(1, 23) = 9.91, MSe = 178, d = 0.86; F_2_(1, 23) = 6.86, MSe = 221, d = 0.79 or the incorrect-low overlap condition, F_1_(1, 23) = 6.81, MSe = 267, d = 0.78; F_2_(1, 23) = 7.22, MSe = 235, d = 0.74. The difference between the two incorrect-overlap conditions was not significant, both Fs < 1.

Recall that Cook [26] found that initial reading times on the target sentence containing an anaphor were a function of the semantic overlap between the anaphor and the antecedent, and that this supported an account in which readers do not fully resolve anaphors before they move on in the text. She argued that it took additional time for information about the antecedent to be reactivated, integrated, and validated against incoming information about the anaphor, such that readers did not experience comparable difficulty in both incorrect conditions until downstream in the time course of processing, when readers had moved on to a subsequent sentence. Since her target sentences were not designed for the kind of fine-grained analyses used in eye tracking studies, it is possible that her effects were distributed across the entire sentence instead of isolated to a single word or short phrase. The results presented here indicate that processing difficulty did not occur on the anaphor itself, but downstream, after readers had already moved on to subsequent text. Effects of the antecedent condition were observed only in measures that reflected delayed processing (i.e., second-pass, probability of regressions into the anaphor). This highlights the importance for inclusion of measures that reflect different points in the time course of processing. Moreover, the usage of a self-paced line by line paradigm did not allow participants to regress to previously encountered content from the passage. The question remains, though, whether they would have done so if the entire text had been available. That is, if participants do regress back to earlier information in a text to resolve encountered inconsistencies, how far back in the text do they go? 

In order to answer that question, we next turned to an exploratory analysis of the overall patterns of regressions in the text out of the target region. As mentioned previously, there have been several studies examining overall reading patterns in extended discourse [28,29,30], but those studies either investigated general reading strategies and/or used expository texts. The present experimental context is different in that narrative texts were used to measure processing in a normal comprehension task, and each text contained a one-word anaphoric reference in the target line that was specifically designed to evoke processing difficulty. Thus, we can use the anaphor as a starting point to gain information about how far back in the text readers will go to reread. To our knowledge, this exploratory analysis of general regression patterns has not been presented in the research literature. 

When considering regressions made from the target line across all participants and items, readers made more regressions between words than across lines; on average, participants regressed 1.88 words (SD = 3.86), and 0.08 lines (SD = 0.42) back in the text. A frequency analysis of participants’ regression behaviors revealed that readers made regressions from content in the target line approximately 84.4% of the time. However, 78.3% of all regressions were between only one and four words back in the text. Consistent with this, when examining regressions within versus across lines, 94.1% of regressions were to material within the same line rather than to text on preceding lines. The mean number of words and lines regressed as a function of passage condition appear in Table 3. Note that these exploratory analyses are based only on regressions launched from the target line. Since not all participants made regressions from the target line in each item or condition, our analyses are tested only against error terms based on participants’ variability. 

*Number of Words Regressed from Target*. There was a significant main effect of condition on the number of words regressed from the target region, F(2,52) = 5.15, MSE = 2.73, *p* = 0.01, partial η^2^ = 0.17. Planned comparisons demonstrated that the difference between the correct and incorrect-high overlap conditions was not significant, t(26) = −1.28, *p* = 0.21. However, participants regressed back fewer words from the target in the correct condition than in the incorrect-low overlap condition, t(26) = −2.57, *p* = 0.02, d = 0.73, and they regressed back fewer words in the incorrect-high overlap condition than in the incorrect-low overlap condition, t(26) = −2.07, *p* = 0.049, d = 0.55. 

*Number of Lines Regressed from Target*. The same pattern appeared when the number of lines regressed from the target was analyzed, F(2, 52) = 4.29, MSE = 0.02, *p* = 0.02, partial η^2^ = 0.14. The difference between the correct and incorrect-high overlap conditions, although in the right direction, was not significant, *p* > 0.1. However, the contrast for the correct and incorrect-low overlap conditions was significant, t(26) = −2.48, *p* = 0.02, d = 0.7, but the difference in number of lines regressed from the target in the incorrect-high and incorrect-low overlap conditions did not reach criteria for significance, t(26) = 1.73, *p* = 0.096, d = 0.5. 

This experiment demonstrated that, at least in some discourse processing studies, effects of interest may not appear immediately upon encoding a target region; instead, effects may be observed across a wider time course of processing. In the case of anaphoric processing, effects did not appear until after the reader had already moved past the anaphor in the target sentence, supporting the argument that processing of anaphors may be incomplete, even when readers move on in the text [21,22]. Effects of antecedent condition appeared on regressions into and rereading of the anaphor, as well as in regressions back to previously read content. The purpose of the more exploratory analysis was to examine, when readers do have the opportunity to regress back to previously encountered content in the text, just how far back will they go? The answer is: not very far. When the entire text was available for rereading, readers rarely regressed back more than a line or two–meaning they rarely regressed back to the antecedent itself in order to resolve difficulty caused by an incorrect anaphor. Thus, resolution of difficulty due to incorrect anaphors had to be based on information reactivated from memory either when the anaphor was encountered, or soon thereafter. These eye tracking results, in combination with previous work on anaphor processing [16,17,18], and the previous findings of Cook [25] and Rayner and colleagues [27], support a view in which higher level comprehension of text results from a continuous process of integrating incoming information with and validating it against information that has been reactivated from memory [31,32,33]. 

## 4. Discussion

Given our observation that readers resolve processing difficulty during discourse comprehension without extensive rereading of earlier portions of the text, it must be that reading times on the target sentence reflect difficulty in integrating incoming information and evaluating it against information that has been reactivated from memory. This is the same argument made by researchers who use line-by-line self-paced reading paradigms in which it is not possible for readers to regress back to previous portions of the text (e.g., [15,31]). In anaphor resolution in particular, this allows readers to connect anaphors with distant antecedents without engaging in extensive rereading or experiencing large coherence breaks. The downside of this, however, is that comprehension relies on algorithmic processes that are not perfect in nature. In the present case and in earlier work by Cook [26] and by Rayner and colleagues [27], time to process incorrect anaphors was a function of their overlap with the antecedent, instead of based on whether the anaphor was a correct referent or not. Indeed, Klin and colleagues [34,35,36] argued that in some cases, readers may never fully activate the specific lexical item for an antecedent and instead rely on a partially activated set of conceptual features about the antecedent during the initial stages of anaphor resolution. If the reactivated content is “good enough” [24,25,37], comprehension proceeds. However, as demonstrated here and in Cook’s [26] original study, additional information may become available and lead to processing difficulty downstream of the anaphor. Since Klin and colleagues’ [34,35,36] studies used a single response probe paradigm, they were not able to observe the continuum of processing that the use of eye tracking allowed for here and in Rayner’s [27] study. In general, the results reported here add to a growing body of literature that supports a view in which information is continually being reactivated from memory, integrated with incoming content, and validated with respect to the information in active memory [31,32,33].

The benefit of eye tracking studies beyond line-by-line self-paced reading paradigms, then, is not in the kinds of phenomena that can be studied but in the level of analysis that can be obtained. Eye tracking allows for a more fine-grained measure of where in the time course of reading processing difficulty occurs and what information readers may utilize to resolve that difficulty. This is particularly important when examining processing of particular words or phrases that play out over time. The study reported here focused on the time course of anaphor resolution, demonstrating that resolution is based on the “goodness of fit” between the anaphor and reactivated information, that it continues after readers have moved past the anaphor in the text, and that resolution depends on reactivated information rather than direct access in the text to the previously encountered content. 

This ability to examine processing over time in higher level of comprehension is important, because reliance on measures that fail to examine the full time course of processing may paint a misleading picture of the comprehension process. In another study, Cook and colleagues [38] used eye tracking with Moses Illusion items in which highly related, but incorrect target concepts were embedded in general knowledge statements (e.g., “It was Moses who took two animals of each kind on the Ark). Consistent with previous eye tracking studies on semantic anomalies [39,40,41], Cook and colleagues [38] demonstrated that readers incorrectly responded “true” to illusion statements and did not have any differences in initial reading times (i.e., first fixation duration, first-pass duration) between correct and incorrect content. However, different from previous studies, Cook and colleagues [38] also found that relatively late measures of reading on the target (i.e., regressions and second-pass duration) showed that readers spent more time reprocessing incorrect targets than correct ones, even if they had initially failed to detect the incorrect information and responded “true” to the item. This suggests that participants’ explicit responses to incorrect content in text may not be reflective of the extent to which that information is actually processed. 

The previous paragraphs presented examples of how eye tracking can be used to examine critical issues in higher level comprehension—particularly processing of inconsistent or difficult content in text. In the course of this discussion, we want to revisit our earlier discussion of the importance of careful stimulus design in eye tracking studies of discourse processing. As illustrated in the analysis presented in this article, it is useful to construct text-level stimuli such that comprehension of a very specific region of text (i.e., a target word or phrase) is dependent upon previous portions of text. This allows researchers to understand how comprehension of information may change as a function of the preceding content, even if that content appeared several sentences or lines back in the text. For example, even though studies of lexically ambiguous words (e.g., “bank”) may be focused on lexical access, which is a lower, word-level process, researchers have studied how access of word meaning is influenced by discourse level variables. Wiley and Rayner [42] investigated how processing time on ambiguous words embedded in paragraph length passages was influenced by passage titles as well as passage context. Similarly, Colbert-Getz and Cook [13] examined: (1) whether elaboration of passage context that supported the subordinate meaning of a lexically ambiguous word would influence word processing time; and (2) whether a prior encounter of an ambiguous word in its subordinate sense would influence subsequent processing of the same word in its dominant sense. In both studies just described, the target regions consisted of a lexically ambiguous word and a disambiguating word or phrase; processing of these regions depended upon the preceding passage context. Although there have been fewer studies in which researchers studied the influence of discourse context on sentence processing, researchers use the same general stimulus design strategy. Processing of a particular word or phrase depends on how it is parsed, and parsing may be influenced by the global passage content [43]. Across these studies, though, measures are typically limited to data taken from the target line itself. The results from the present study illustrate why: readers do not appear to reread distant portions of the text to resolve comprehension difficulty, even when those earlier portions of the text are still present on the screen and, thus, available to the reader. For a more detailed discussion of stimulus design issues for eye tracking studies in reading research, see Cook and Wei [11].

Of greatest relevance to the present discussion, though, are studies in which processing of information in a target sentence is dependent upon readers making connections between the target information and previously encountered information. Although the general design of the target region may be the same, the types of questions that are asked may be different. Researchers interested in discourse processing are generally focused on how a developing representation of a text in memory influences processing over time. For example, do readers activate inferences based on preceding contextual information, and do they instantiate those inferences into the evolving discourse model in long-term memory? O’Brien and colleagues [19,20] found that processing times on words that were only implied in a text were just as fast as when those same words had been explicitly mentioned, indicating that the implied concepts had been inferred during reading (i.e., activated; see [23,44,45]) and instantiated into the representation of the text in memory. 

As illustrated in the study in this article, eye tracking studies of discourse can also reveal how processing plays out over time. Although researchers interested in word- and sentence-level processing also examine the time course of processing, what researchers mean by “early” and “later” processing differs across levels of processing. For example, word-level researchers may examine early recognition processes related to orthography and phonology, followed by later processes of semantic access or integration with sentence context. In contrast, discourse processing researchers are generally focused on the time course of encoding new information, linking it with the current contents of active memory, and verifying it against information in long-term memory, e.g., [31,32,33]. Since passage-level texts contain more information that can be held in the reader’s working memory, readers must rely on information that is reactivated from long-term memory. Although this may include information previously presented within the text, as in the examples described in the preceding paragraph, it may also include information that is reactivated from the reader’s general world knowledge, or semantic memory. Thus, in eye tracking studies of higher level comprehension, “early” processing may reflect influences of content that is active when a target word is encoded, whereas “later” processing may reflect influences of information that is not activated and incorporated into the ongoing discourse representation until the reader has already moved past the target in the text. 

This interpretation of “early” and “late” influences in comprehension has been applied to studies of the time course of influences of previously encountered contextual content versus information from general world knowledge on processing of incoming information. Using eye tracking technology, Garrod and Terras [46] examined whether readers’ processing of role fillers was initially influenced by either information from general world knowledge or the previous discourse context. They had participants read short texts in which the target region indicated either an appropriate or inappropriate role filler (based on general world knowledge) for an action presented in a previous sentence. For example, the target phrase “the pen dropped” is an appropriate role filler for the preceding sentence “The teacher was busy writing a letter of complaint to a parent” but is inappropriate if the preceding sentence was “The teacher was busy writing an exercise on the blackboard.” Eye tracking measures revealed no initial effect of appropriateness on processing the noun, “pen.” However, times in the region of the verb, “dropped,” and regressions from it back to the noun “pen” indicated delayed processing difficulty when the pen was an inappropriate role filler for the preceding action. Garrod and Terras argued that early processing of the role filler represented low-level associative bonding of the role filler (pen) to the preceding action (writing), but this link was subsequently resolved against the broader discourse context (writing a letter vs. writing on the chalkboard). Thus, processing difficulty due to a mismatch between the role filler and the context was not observed until relatively late in the time course. 

Cook and Myers [47] extended this work by creating scripted narrative texts (e.g., a rock band context) in which the initial encounter with a role filler was either appropriate (a song was played by a guitarist) or inappropriate (a song was played by the manager) with respect to general world knowledge. Consistent with Garrod and Terras’ [46] findings, processing times were a function of the appropriateness of the role filler for the action described. The passage continued, however, and a second encounter with the role filler was also either appropriate or inappropriate with respect to general script-based knowledge. More important, though, the second encounter either matched or mismatched the first encounter. Cook and Myers found that when the second encounter matched the first encounter, regardless of whether it was appropriate or not, initial processing of this encounter was facilitated. Subsequent processing on the second encounter, though, showed a delayed effect of appropriateness of the role filler; readers had increased regressions and longer second pass reading times for the inappropriate role fillers. Cook and Myers argued that the early effects of appropriateness on the first encounter, but the delayed effects of appropriateness on the second encounter, suggested that either general world knowledge or context has the potential to be reactivated and influence initial processing of incoming information. However, as additional information continues to be reactivated, it has the potential to influence processing downstream in the time course, even if the reader has moved on in the text. Although one source of knowledge may dominate early processing of target content, the fine-grained nature of eye tracking measures allow researchers to examine the extent to which the other sources of knowledge come into play downstream. 

The argument that initial processing is influenced by the winner of a “race” for activation between contextual information and general world knowledge is consistent with assumptions of the RI-Val model of discourse comprehension proposed by Cook and O’Brien [31,32,33]. They argued that comprehension can be explained in terms of three parallel asynchronous stages of processing that each operate on the output of the preceding stage. In the first stage (R), information is reactivated from long-term memory in response to incoming content via a passive retrieval mechanism, e.g., [48,49], and this includes both previously read content as well as information from general world knowledge. As soon as information becomes available, it is linked to, or integrated (I), with the contents of working memory on the basis of goodness of fit in the second stage. The third stage involves validating (Val) linkages against the contents of active memory via a feature-based partial matching mechanism [50,51,52]. These stages are assumed to be passive in nature and, thus, run to completion; they are also continuously operating. Thus, new information may be reactivated even as the validation stage is starting. This is true regardless of whether readers have reached their coherence threshold, the point in time at which attention shifts to new information in the text. This means that new information may still be coming available in working memory even after the reader has moved on in the text. Since processing operates on either side of the coherence threshold, it is possible to observe processing difficulty either immediately upon encountering the problematic content, or after a delay. 

Cook [26] used the RI-Val model to explain her finding that early processing of anaphors was based on goodness of fit; as contextual information about the antecedent continued to become available in memory, however, that content influenced processing downstream from the anaphor. Although Cook’s results were based on line-by-line self-paced reading data, the same general pattern of results was found with the eye tracking data reported here; incorrect anaphors resulted in processing difficulty, but only in measures that reflected processing relatively late in the time course (i.e., regressions, second-pass duration). The present findings also show, though, that readers did not utilize the entirety of the text to resolve that processing difficulty; most regressions were within the same line, and there were relatively few regressions more than one or two lines back in the text—not far enough to reread the portion of the text containing the explicit mention of the antecedent. As suggested previously, this means that processing difficulty was resolved based on the information that had been reactivated in memory. Given the continuous nature of processing assumed by the RI-Val model, information about the antecedent becomes available in working memory over time, meaning that early processing of an anaphor may be based on incomplete content. Resolution continues as more information becomes available, and this may continue occur even after the reader has moved on in the text. 

In another discourse processing study, Creer, Cook, and O’Brien [53] examined how narrator perspective (i.e., first-person, third-person) influenced processing of spatial inconsistencies embedded in texts. Across multiple self-paced line-by-line experiments, they found that under normal reading conditions, readers were disrupted by spatial inconsistencies involving the protagonist when texts were written in the first-person perspective, but not when they were written in the third-person perspective. Creer et al. argued that the disruption was due to readers having difficulty validating incoming content against information reactivated from the discourse representation in long-term memory. Consistent with the view that validation occurs relatively late in the time course of processing, an eye tracking experiment isolated the inconsistency effects to measures that reflected processing that occurred after participants had initially encountered the inconsistent content (e.g., go-past duration, second-pass duration). 

Although the present study demonstrated that readers do not typically regress very far in the text to reread information that may help in resolving inconsistencies, it may be possible to push them to do so by increasing their coherence threshold, within the assumptions of the RI-Val model [32,33]. Recent studies have demonstrated that subtle changes to the study procedure can result in large shifts in the reader’s coherence threshold. For example, Williams and colleagues [54] argued that changing the number of comprehension questions asked at the end of each passage may shift the coherence threshold, such that readers will either wait more or less time for validation processes to complete before they move on in the text. When comprehension questions were increased, the coherence threshold was high, meaning that the validation process had more time to complete before readers moved on to subsequent text (see also [53]). When comprehension questions were decreased, the coherence threshold was low, and readers waited very little time for validation to complete before moving on to subsequent information. Within the present study context, it is possible that shifting the coherence threshold with similar manipulations would alter the extent to which readers experience difficulty validating the incorrect anaphor. By this logic, within the present study context, a higher coherence threshold would result in more efforts to validate the incorrect anaphor before readers move on to subsequent information, possibly leading to more regressions back to previous text, including the antecedent. Additionally, a lower coherence threshold may reduce the extent to which readers attempt to validate the anaphor before moving on in the text, possibly reducing difficulty due to incorrect anaphors altogether. 

The distribution of processing effects over time is a growing area of interest in discourse comprehension research. This area of research is uniquely suited to paradigms and measures that allow for observation of the time course of processing effects—such as eye tracking. Even before the positing of theoretical models of discourse comprehension in which the timing of effects is critical (e.g., RI-Val), we have long argued for the importance of using measures that allow more than a single window into processing. This is now more important than ever. As tests of theoretical assumptions in discourse comprehension research hinge on which sources of information influence processing and when, it is essential that researchers utilize measures that provide a wider view of the time course of processing. Although several studies have accomplished this with careful development and presentation of stimuli in line-by-line self-paced reading paradigms, the use of eye tracking technology can complement that work by providing finer-grained analyses that allow researchers to isolate effects to critical words or phrases and to determine how they are processed over time. However, we want to end with a note of caution–researchers should be careful not to equate specific measures with specific processes. As Cook and Wei [11] argued, the considerable overlap among measures makes mapping specific measures onto specific cognitive processes a complex and unwise task. Instead, we recommend the approach long recommended by Rayner [8], in which researchers use a variety of convergent measures that cover a range of points on the temporal continuum of processing.

## Figures and Tables

**Table 1:** Sample passage from Cook [26], modified for the eye tracking study.

***Correct Antecedent Condition***
Terry and her friend Jill drove to a music shop. As they entered the store, Terry saw a beautifulcello. The large instrument was almost bigger than she was. Terry decided she would teach herself how to play it. She imagined herself sitting down to play the heavy instrument. After thinking for a few minutes, she decided to buy it. Just then, Jill walked over to where Terry was standing. Terry showed Jill the ***cello*** she had bought at the store that day. She even tried to play a few notes.
***Incorrect-High Overlap Condition***
Terry and her friend Jill drove to a music shop. As they entered the store, Terry saw a beautiful violin. The small instrument fit perfectly between her chin and shoulder. Terry decided she would teach herself how to play it. She imagined herself dancing as she playedthe lightweight instrument. After thinking for a few minutes, she decided to buy it. Just then, Jill walked over to where Terry was standing. Terry showed Jill the ***cello*** she had bought at the store that day. She even tried to play a few notes.
***Incorrect-Low Overlap Condition***
Terry and her friend Jill drove to a music shop. As they entered the store, Terry saw a beautifuloboe. The keys were bright and shiny, and the case was lined in black velvet. Terry decided she would teach herself how to play it. She imagined herself fingering the keys tocreate perfect notes. After thinking for a few minutes, she decided to buy it. Just then, Jill walked over to where Terry was standing. Terry showed Jill the ***cello*** she had bought at the store that day. She even tried to play a few notes.

**Table 2:** Mean (and standard deviations) for first-pass duration, go-past duration, and second-pass duration (in milliseconds), with probability of regressions into and out of the anaphor, as a function of antecedent condition.

	Antecedent Condition		
Measure	Correct	Incorrect-High Overlap	Incorrect-Low Overlap
First-pass duration	274 (48.98)	279 (52.91)	283 (46.53)
Go-past Duration	367 (201.79)	341 (68.75)	378 (111.45)
Second-pass duration	15.75 (35.42)	45.58 (50.45)	69.42 (78.75)
Probability of Regression out of the Anaphor	0.17 (0.19)	0.17 (0.2)	0.20 (0.18)
Probability of Regression into Anaphor	0.02 (*0.05*)	0.11 (*0.13*)	0.11 (*0.15*)

**Table 3:** Mean number of words and lines regressed (and standard deviations) from the target region as a function of antecedent condition.

	Antecedent Condition		
Measure	Correct	Incorrect-High Overlap	Incorrect-Low Overlap
Number of Words Regressed from Target	1.21 (0.68)	1.48 (1.11)	2.57 (2.56)
Number of Lines Regressed from Target	0.01 (0.04)	0.04 (0.09)	0.13 (0.24)
